# Protective effect of aqueous extract of *Embelia ribes* Burm fruits in middle cerebral artery occlusion-induced focal cerebral ischemia in rats

**DOI:** 10.4103/0253-7613.44153

**Published:** 2008-10

**Authors:** Uma Bhandari, M. Nazam Ansari

**Affiliations:** Department of Pharmacology, Faculty of Pharmacy, Hamdard University, New Delhi, India

**Keywords:** Cerebral ischemia, *Embelia ribes*, middle cerebral artery occlusion, oxidative stress

## Abstract

**Objective:**

The present study was carried out to evaluate the neuroprotective effect of the aqueous extract of *Embelia ribes*, in focal ischemic brain.

**Materials and Methods:**

Adult male Wistar albino rats were fed with the aqueous extract of *Embelia ribes* (100 and 200 mg/kg, p.o.) for 30 days. After 30 days of feeding, all the animals were anaesthetized with chloral hydrate (400 mg/kg, i.p.). The right middle cerebral artery was occluded with a 4-0 suture for 2 h. The suture was removed after 2 h, to allow reperfusion injury. The animals were used for grip strength measurement, biochemical estimation in serum and brain tissue (hippocampus and frontal cortex) and cerebral infarct size measurement.

**Results:**

In the ischemic group, a significant (*P* < 0.01) alteration in the markers of oxidative damage (thiobarbituric acid reactive substances (TBARS); reduced glutathione (GSH); glutathione peroxidase (GPx); glutathione reductase (GR); and, glutathione-S-transferase (GST)) was observed in the hippocampus and frontal cortex, as compared to sham operated rats. We observed that the animals treated with the aqueous extract of *Embelia ribes* had a significant (*P* < 0.01) increase in the poststroke grip strength activity. Further, supplementation with aqueous extract of *Embelia ribes* reversed the levels/activities of the above mentioned biochemical parameters significantly (P< 0.01) and also resulted in decreased cerebral infarct area, as compared to the ischemic group.

**Conclusion:**

The results of our study, for the first time, provide clear evidence that aqueous extract of *Embelia ribes* pretreatment ameliorates cerebral ischemia/reperfusion injury and enhances the antioxidant defense against middle cerebral artery occlusion-induced cerebral infarction in rats; it exhibits neuroprotective property.

## Introduction

Being the second most common cause of death in industrial countries and one of the major causes of death and disability, stroke has a great effect on public health. Among the neurological diseases, stroke accounts for the largest number of hospitalizations.[1] A variety of mechanisms are involved in ischemic brain injury. The cascade of events leading to neuronal injury and death in ischemia includes the release of cytokines, free radicals, and platelet activation.[2] Furthermore, reactive oxygen species, including the superoxide anion, hydroxyl radical, and peroxynitrite radical, have been implicated in neuronal cell damage and death after cerebral ischemia.[3] Brain ischemia induces excessive release of excitatory amino acids and subsequent receptor activation, leading to calcium influx, metabolic and electrophysiological dysfunction, lipid peroxidation, and other oxidative events.[4] Increasing evidence has indicated that ischemia/reperfusion, which occurs either due to blockage of the Middle Cerebral Artery (MCA) or due to recirculation of blood flow, causes oxidative stress that may potentiate ischemic injury.[5] Therefore, inhibition of production and enhanced degradation of reactive oxygen species with pharmacological agents have been found to limit the extent of brain damage after stroke-like events[6] and antioxidants have been the focus of studies for developing neuroprotective agents to be used in stroke therapy. Many antioxidants have been developed in *in vitro* and *in vivo* experiments and some of these have been tested in clinical studies of stroke.[7]

The lack of effective and widely applicable pharmacological treatments for ischemic stroke patients may explain a growing interest in traditional medicines, for which extensive observational and anecdotal experience has accumulated over the past years. It has been suggested that some herbal medicines, or their products, may improve microcirculation in the brain,[8] protect against ischemic reperfusion injury,[8] possess neuroprotective properties[9] and inhibit apoptosis,[10] thus justifying their use in ischemic stroke patients.

*Embelia ribes* Burm (Myrsinaceae), commonly known as Vidanga, is a large woody climbing shrub and is widely distributed throughout India. It is highly esteemed in Ayurveda as a powerful anthelmintic.[11]*Embelia ribes* is also reported to have antifertility action.[12] Analgesic property was reported for embelin and its derivatives.[13] The plant is used as an anti-inflammatory drug to relieve rheumatism and fever.[14] The fruit cures tumors, ascites, bronchitis, jaundice and mental disorders.[15] Bhandari *et al.*[16,17] have reported the antidiabetic, antidyslipidemic and antioxidant activity of *Embelia ribes* Burm in streptozotocin-induced diabetes in rats, using gliclazide as the positive control drug. Recently, Bhandari *et al.*[18] have reported the cardioprotective activity of the aqueous extract of *Embelia ribes* in isoproterenol induced myocardial infarction in albino rats.

Middle cerebral artery occlusion (MCAO) is most commonly model used to induce experimental focal cerebral ischemia.[19] The advantages of the MCAO model are its reproducibility and ease of reperfusion. Besides, the type of ischemic injury observed is similar to that found in human.[20]

The objective of the present study is to induce focal cerebral ischemia by MCAO and to investigate the neuroprotective potential of the aqueous extract of *Embelia ribes* in MCAO-induced focal cerebral ischemia in albino rats, using biochemical markers and cerebral infarct size measurement.

## Materials and Methods

### Chemicals

Triphenyl tetrazolium chloride (TTC) dye used in the study was obtained from Sigma chemicals (St Louis, MO, USA). All other chemicals used were of analytical grade. Double distilled water was used for all biochemical assays.

### Preparation of the aqueous extract of Embelia ribes

Dried fruits of *Embelia ribes* Burm were purchased from the local market, New Delhi, India, in August 2006 and botanical authentification was carried out by the Department of Botany, Faculty of Science, Hamdard University, New Delhi, India (voucher specimen no. UB 2). The dried and coarsely powdered drug (100 g) was packed in a soxhlet apparatus.

Water (300 ml) was placed in a round bottom flask and a reflux condenser was attached above the soxhlet. The water was heated to boil on heating mantle and was subjected to extraction for 72 hours (thrice, 24 h each time). The filtrate was evaporated under a vacuum drier (Narang Scientific Works Pvt. Ltd., New Delhi, India) and the brown mass residue obtained was stored at 4^°^ C for further use. The average yield of the aqueous extract of*Embelia ribes* was approximately 5.261%. The aqueous extract of *Embelia ribes* (ER) – 100 and 200 mg/kg body weight – was dissolved in 1% Tween 80 in distilled water and administered to adult male Wistar albino rats by oral route, since earlier studies reported the effectiveness of *Embelia ribes* extracts in doses of 100 and 200 mg/kg body weight.[18]

### Standardization of the aqueous extract of Embelia ribes

Preliminary phytochemical screening of the aqueous extract of *Embelia ribes* fruits was carried out for the detection of phytoconstituents, using standard chemical tests. Alkaloids, carbohydrates, phenolic compounds, flavonoids, proteins and saponins were detected in the extract. Further, high performance thin layer chromatography (HPTLC) fingerprints of the aqueous extract was established using CAMAG HPTLC (WinCAT software, version 2.2) and benzene: ethyl acetate (6: 4) as solvent system, which showed the presence of seven spots (Max R_f_ values: 0.32, 0.34, 0.42, 0.45, 0.52, 0.65 and 0.78) at 520 nm wavelength. It can, thus, be concluded that the antioxidant effect of *Embelia ribes* can be due to the presence of alkaloids, flavonoids, phenolic compounds and saponins.

### Animals

The experimental protocol was approved by the Institutional Animal Ethics Committee (IAEC) of Hamdard University, New Delhi, which is registered with the Committee for the Purpose of Control and Supervision of Experiments on Animals (CPCSEA), Government of India, (Registration no. 173/CPCSEA, dated 28 January, 2000). Healthy male adult Wistar rats (200-250 g), procured from the Central Animal House Facility, Jamia Hamdard, New Delhi, and acclimatized under standard laboratory conditions at 25 ± 2°C, relative humidity (50 ± 15 %) and normal photoperiod (12 h light dark cycle) for seven days, were used for the experiment. The animals were fed with commercial rat pellet diet (manufactured by Nav Maharashtra Chakan Oil Mills Ltd, Delhi, India) and water ad libitum. Adequate measures were taken to minimize pain or discomfort and the experiments were conducted in accordance with the international standards on animal welfare; the measures were also compliant with local and national regulations.

### Middle cerebral artery occlusion

The right middle cerebral artery occlusion (MCAO) was performed using an intraluminal filament model and the method described by Longa *et al.*[21] In brief, the rats were anesthetized with chloral hydrate (400 mg/kg, i.p.), a 4-0 nylon monofilament (Ethicon, Johnson and Johnson Ltd., Aurangabad, India) with a blunt end was introduced into the external carotid artery (ECA) and advanced into the middle cerebral artery via the internal carotid artery (ICA) (17-20mm), until a slight resistance was felt. Such resistance indicated that the filament had passed beyond the proximal segment of the anterior cerebral artery (ACA). At this point, the intraluminal suture blocks the origin of the MCA and occludes all sources of blood flow from the internal carotid artery, ACA and the posterior cerebral artery. Two hours after the induction of ischemia, the filament was slowly withdrawn and the animals were returned to their cages for a period of 22 hous of reperfusion. Throughout the procedure, the body temperature was maintained at 37^°^ C, with a thermostatically controlled infrared lamp. In sham rats, the ECA was surgically prepared for the insertion of the filament, but the filament was not inserted.

### Animal protocol

Male Wistar rats (200-250g) were randomly divided into six experimental groups (n = 10 each). The first group served as sham and 1% Tween 80 in distilled water was given orally for 30 days. The second and third groups were sham rats that had been pretreated with ER 100 mg/kg and 200 mg/kg p.o. respectively for 30 days. The fourth group was the MCAO group in which ischemia was induced for 2 h, followed by reperfusion for 22 h. The fifth and sixth groups were treated by ER 100 mg/kg and 200 mg/kg p.o. respectively for 30 days, followed by MCAO induced cerebral ischemia.

After 24 h of the lesioning, grip strength in all the animals was measured, using the grip strength meter (Panlab, Barcelona, Spain) for evaluation of neuromuscular strength, as described by Ali *et al.*[22] After grip strength measurement, blood samples were drawn from the tail vein from all the groups and serum was separated for biochemical estimations. Thereafter, the animals were sacrificed by cervical dislocation and their brains were taken out quickly for infarct size measurement. Then hippocampus and frontal cortex was dissected for biochemical estimations. The hippocampus and frontal cortex were homogenized in 10 mM phosphate buffer (PB, pH 7.4). The homogenate was used for estimation of lipid peroxidation in terms of TBARS content, and centrifuged at 800 × *g* for 5 min at 4°C, to separate the nuclear debris. The supernatant was further centrifuged at 10,000 × *g* for 20 min at 4°C, to get the postmitochondrial supernatant (PMS), which was used for the estimation of reduced GSH and antioxidant enzymes activity.

### Biochemical analysis

In serum, lactate dehydrogenase (LDH) was estimated using a method described by Lum *et al.*[23] A 10% homogenate of brain tissues (frontal cortex and hippocampus) in phosphate buffer was used for the assay of the malondialdehyde, according to the method described by Utley *et al.*[24] and modified by Islam *et al.*[25] The PMS (frontal cortex and hippocampus) was used for the assay of glutathione (GSH) content,[26] glutathione peroxidase (GPx) activity,[27] glutathione reductase (GR) activity[28] and glutathione S transferase (GST) activity.[29]

### Measuring the size of the cerebral infarct

The brains were sectioned coronally (thickness – 2 mm) and stained with 1% 2, 3, 5-triphenyl tetrazolium chloride (TTC) in phosphate buffer (0.1M, pH 7.4) for 30 min at 37^°^ C, as described by Joshi *et al.*[30] The sections were refrigerated in 4% formaldehyde in phosphate buffer for 30 min and the image of each section was taken.

### Statistical analysis

The data are expressed as mean±SEM. Statistical differences between means were determined by one-way analysis of variance (ANOVA), followed by Dunnett t-test. The values of *P* < 0.01 were considered as significant.

## Results

The effect of aqueous extract of *Embelia ribes* on grip strength

The basal grip strength was found to be 0.942 ± 0.006 kg units. A significant (*P* < 0.01) decrease in the grip strength was observed in the ischemic (MCAO) group, as compared to the sham rats. Aqueous extract of *Embelia ribes* treated rats showed a significant (*P* < 0.01) increase in grip strength, as compared to the ischemic (MCAO) group. However, no significant change in grip strength was observed in the rats treated only with the aqueous extract of *Embelia ribes*, as compared to the sham rats [Table 1].

**Table 1:** Effect of aqueous extract of *Embelia ribes* on grip strength and serum LDH levels in MCAO-induced focal cerebral ischemia in rats

*Groups*	*Grip strength (Kg Units)*	*LDH (IU/L)*
Sham	0.942 ± 0.006	74.356 ± 3.493
ER-100 mg/kg + sham	0.945 ± 0.011	73.934 ± 1.308
ER-200 mg/kg + sham	0.931 ± 0.007	71.909 ± 5.100
MCAO	0.645 ± 0.005^a^	190.406 ± 5.946^a^
ER-100 mg/kg + MCAO	0.954 ± 0.004^b^	119.848 ± 10.136^b^
ER-200 mg/kg + MCAO	0.958 ± 0.001^b^	114.615 ± 2.899^b^

Values are mean ± SEM (n=10)

a *P*<0.01, as compared to the sham group

b *P*<0.01, as compared to the MCAO group

### Effect of aqueous extract of Embelia ribes on serum LDH levels

The basal serum LDH levels were found to be 74.356 ± 3.493 IU/L. A significant (*P* < 0.01) increase in the activity of LDH in serum was observed in the ischemic (MCAO) group rats, as compared to the sham group; whereas, aqueous extract of *Embelia ribes* treatment significantly (*P* < 0.01) resulted in decreased serum LDH levels when compared with the ischemic (MCAO) group rats. However, no significant changes in LDH activity were observed in the rats treated only with the aqueous extract of *Embelia ribes*, as compared to the sham rats [Table 1].

### Effect of aqueous extract of Embelia ribes on TBARS levels

TBARS level in frontal cortex and hippocampus were found to be significantly (*P* < 0.01) higher in the ischemic (MCAO) group, as compared to the sham rats, while aqueous extract of *Embelia ribes* treatment significantly (*P* < 0.01) decreased these elevated levels when compared with the ischemic (MCAO) group rats. However, no significant changes in TBARS levels were observed in the rats treated only with aqueous extract of *Embelia ribes*, as compared to the sham rats [Table 2].

**Table 2:** Effect of aqueous extract of *Embelia ribes* on thiobarbituric acid reactive substances levels in MCAO-induced focal cerebral ischemia in rats

*Groups*	TBARS (nmol TBARS formed/30 min/mg protein)	
	
	Hippocampus	Cerebral cortex
Sham	2.009 ± 0.010	3.070 ± 0.036
ER-100 mg/kg + sham	1.988 ± 0.001	3.043 ± 0.021
ER-200 mg/kg + sham	1.984 ± 0.008	3.018 ± 0.006
MCAO	7.460 ± 0.101^a^	8.492 ± 0.241^a^
ER-100 mg/kg + MCAO	3.903 ± 0.008^b^	4.875 ± 0.008^b^
ER-200 mg/kg + MCAO	3.260 ± 0.022^b^	4.268 ± 0.011^b^

Values are mean ± SEM (n=10)

a *P*<0.01, as compared to the sham group

b *P*<0.01, as compared to the MCAO group

### Effect of aqueous extract of Embelia ribes on GSH, GPx, GR and GST levels

As a result of cerebral ischemia for two hours, followed by reperfusion, a significant (*P* < 0.01) reduction in GSH, GPx, GR and GST content of hippocampus and frontal cortex were observed in the ischemic (MCAO) group, as compared with the sham group rats. This reduction is significantly (*P* < 0.01) reversed by the aqueous extract of *Embelia ribes* treatment, when compared with the ischemic (MCAO) group rats. However, no significant changes in GSH, GPx, GR and GST ** levels of hippocampus and frontal cortex were observed in the rats treated only with the aqueous extract of *Embelia ribes*, as compared to the sham rats [Tables 3 and 4].

**Table 3:** Effect of aqueous extract of *Embelia ribes* on glutathione (GSH) and glutathione peroxidase (GPx) levels in MCAO-induced focal cerebral ischemia in rats

*Groups*	*GSH (nmol GSH/mg protein)*		*GPx (nmol NADPH oxidized/min/mg protein)*	
		
	Hippocampus	Cerebral cortex	Hippocampus	Cerebral cortex
Sham	1.563 ± 0.043	1.816 ± 0.035	59.720 ± 1.885	120.474 ± 5.042
ER-100 mg/kg + sham	1.596 ± 0.073	1.846 ± 0.077	60.723 ± 1.346	121.879 ± 3.130
ER-200 mg/kg + sham	1.597 ± 0.061	1.841 ± 0.061	61.503 ± 1.278	123.547 ± 2.644
MCAO	0.888 ± 0.048^a^	1.132 ± 0.110^a^	23.058 ± 1.989^a^	76.836 ± 5.007^a^
ER-100 mg/kg + MCAO	1.277 ± 0.065^b^	1.479 ± 0.073^b^	35.464 ± 4.020^c^	96.119 ± 4.888^c^
ER-200 mg/kg + MCAO	1.309 ± 0.068^b^	1.535 ± 0.072^b^	38.741 ± 4.071^b^	99.506 ± 4.598^b^

Values are mean ± SEM (n = 10)

a *P*<0.01, as compared to the sham group

b *P*<0.01

c *P*<0.05, as compared to the MCAO group

**Table 4:** Effect of aqueous extract of *Embelia ribes* on glutathione reductase and glutathione-S-transferase (GST) levels in MCAO- induced focal cerebral ischemia in rats

*Groups*	*GR (nmol NADPH oxidized/min/mg protein)*		*GST (nmol CDNB conjugate formed/min /mg protein)*	
		
	Hippocampus	Cerebral cortex	Hippocampus	Cerebral cortex
Sham	44.141 ± 2.491	68.642 ± 2.101	79.583 ± 4.941	46.296 ± 3.152
ER-100 mg/kg + sham	44.917 ± 2.185	69.963 ± 2.254	79.758 ± 2.889	47.021 ± 2.155
ER-200 mg/kg + sham	46.413 ± 1.565	70.221 ± 1.093	81.196 ± 2.181	47.949 ± 2.040
MCAO	20.375 ± 0.899^a^	26.630 ± 2.066^a^	29.838 ± 2.680^a^	21.335 ± 2.225^a^
ER-100 mg/kg + MCAO	33.890 ± 4.074^c^	40.533 ± 4.588^c^	49.924 ± 3.122^b^	37.909 ± 2.729^b^
ER-200 mg/kg + MCAO	37.208 ± 4.125^b^	44.094 ± 4.600^b^	54.753 ± 4.042^b^	38.819 ± 2.745^b^

GR = Glutathione reductase, Values are mean ± SEM (n=10)

a *P*<0.01, as compared to the sham group

b *P*<0.01

c *P*<0.05, as compared to the MCAO group

### Effect of the aqueous extract of Embelia ribes on the infarct size

**Figure 1:**
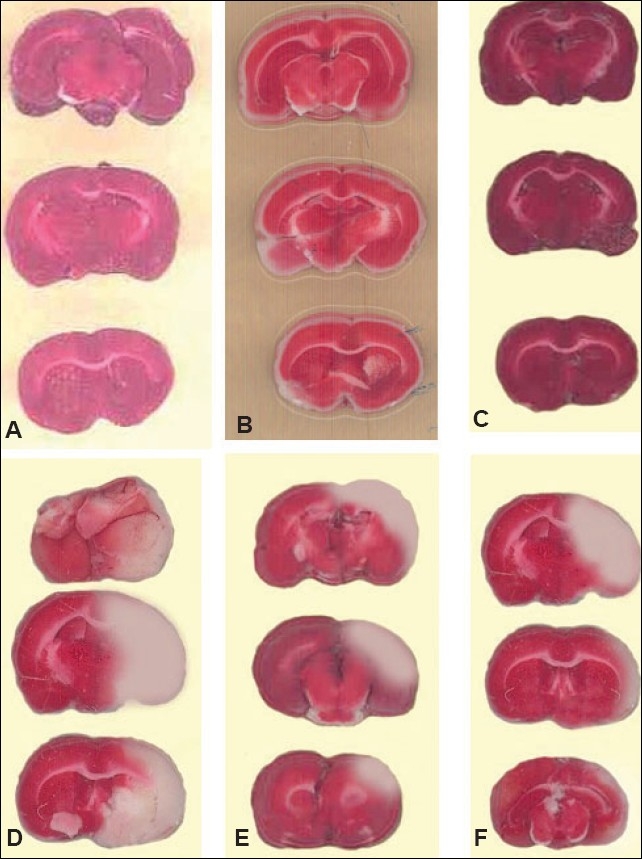
Representative photographs of brain sections showing the neuroprotective effect of the aqueous extract of Embelia ribes on focal cerebral ischemia. (A) Sham group, (B) ER-100 mg/kg + sham, (C) ER-200 mg/kg + sham, (D) MCAO group, (E) ER-100 mg/kg + MCAO and (F) ER-200 mg/kg + MCAO. The dark pink area indicates the normal area while the white area indicates the infarct area.

The infarction in representative sections from different groups is shown in [Figure 1 (A-F)]. In the MCAO group [Figure 1D], a marked infarction (white unstained tissue) was found in the cerebral cortex, as compared to the sham group [Figure 1A]. The pretreatment with the aqueous extract of *Embelia ribes* in ER-100 mg/kg + MCAO and ER-200 mg/kg + MCAO groups [Figure 1E] and [1F] showed significant reduction in the infarct size (pink stained tissue), as compared to the MCAO group.

## Discussion

A great deal of effort has been directed toward searching for a new drug that can be used for protection of cerebral ischemia-reperfusion injury. The results obtained in the present investigation suggest that the aqueous extract of dried fruits of *Embelia ribes* has protective effect against cerebral ischemia-reperfusion injury-induced oxidative stress in a rat model.

A number of processes have been implicated in the pathogenesis of oxygen deprivation-induced cell injury. These include the disturbances of cell calcium homeostasis, depletion of adenine nucleotides, activation of enzymes like phospholipases with production of toxic lipid metabolites, proteases and endonucleases and generation of reactive oxygen species (ROS), which can cause oxidative damage to cellular macromolecules.[31] Increasing evidence has indicated that ischemia/reperfusion occurs due to oxidative stress that may potentiate ischemic injury.[32] Free radicals initiate lipid peroxidation of the membrane bound polyunsaturated fatty acids, leading to impairment of the membrane structural and functional integrity.[33] For these reasons, antioxidants have been the focus of studies for developing neuroprotective agents to be used in stroke therapy.

Free radicals are thought to cause behavioral deficits in experimental animals.[34] In the present study, it is suggested that aqueous extract of *Embelia ribes*, which is a potent antioxidant, has protected neurobeha-vioral deficits of animals by scavenging free radicals. The exact mechanism of this hypothesis is to be explored yet.

Lactate dehydrogenase was measured to evaluate the role of antioxidative stress in the protection of aqueous *Embelia ribes* extract. Twenty-four hours after ischemia/reperfusion injury, significant (*P* < 0.01) rises in LDH was observed in the ischemic rats, as compared to the sham group. In the MCAO group, treatment with the aqueous extract of *Embelia ribes* significantly (*P* < 0.01) decreased the LDH levels, as compared to the MCAO group.

The large numbers of polyunsaturated fatty acids (PUFAs) make cell membranes particularly vulnerable to lipid peroxidation. The oxidation of PUFAs causes them to be more hydrophilic, thereby altering the structure of the membrane with resultant changes in fluidity and permeability. Lipid peroxidation can also inhibit the function of membrane bound receptors and enzymes.[35,36]

We assessed the effect of aqueous extract of *Embelia ribes* on lipid peroxidation, which was measured in terms of MDA, a stable metabolite of the free radical-mediated lipid peroxidation cascade. The MDA levels increased significantly (*P* < 0.001), following cerebral ischemia reperfusion injury. Aqueous extract of *Embelia ribes* reversed the increase of MDA levels to a considerable extent, thereby confirming its antioxidant role in ischemia reperfusion injury.

It has been proposed that antioxidant changes reflect an altered redox balance in several pathological states.[37] In other words, antioxidants would be consumed in the reaction with free radicals. Therefore, the measurement of endogenous antioxidants enzymes i.e. GPx, GR and GST has been performed to estimate the amount of oxidative stress. The activity of endogenous antioxidant enzymes was decreased significantly (*P* < 0.01) in the MCAO group, as compared to the sham group. In the MCAO group, the animals treated with the aqueous extract of *Embelia ribes* produced a significant (*P* < 0.01) increased activity of endogenous antioxidant enzymes.

Reduced glutathione (GSH) is one of the primary endogenous antioxidant defense systems in the brain, which removes hydrogen peroxide and lipid peroxides.[38] Decline in GSH levels could either increase or reflect oxidative status.[39] In our experiment, depletion in GSH was observed in the frontal cortex and hippocampus of the ischemic rats. It has been shown that depletion of GSH in ischemia reperfusion injury can be attributed to several factors such as cleavage GSH to cysteine, decrease in the synthesis of GSH and the formation of mixed disulfides, causing their cellular stores to be depleted.[40] Interestingly, the rats fed with aqueous extract of *Embelia ribes* at both the doses (100 and 200 mg/kg) had increased GSH levels in various brain regions, but the mechanism involved is not known.

This study suggests that aqueous extract of *Embelia ribes* administration to the normal rat did not show any effect on the activity of endogenous antioxidant enzymes and oxidative stress markers in various brain regions of normal rat.

The activity of aqueous extract of *Embelia ribes* appears to work by restoring the altered antioxidants enzymes activity as well as by decreasing the production of lipid peroxides in frontal cortex and hippocampus regions of brain. Interestingly, aqueous *Embelia ribes* extract exerts its antioxidant effect by decreasing LDH, total cholesterol, triglycerides, LDL-C and lipid peroxide levels, which are implicated in cerebral ischemic reperfusion injury. Therefore, on the basis of the present observations of the study, *Embelia ribes* could be an important herbal drug for neuroprotection.
